# Small Animal Orthopedic Surgery, Physical Therapy and Rehabilitation

**DOI:** 10.3390/ani15030351

**Published:** 2025-01-25

**Authors:** L. Miguel Carreira, J. C. Alves

**Affiliations:** 1 Anjos of Assis Veterinary Medicine Centre—CMVAA, Rua D.^a^ Francisca da Azambuja n° 9-9A, 2830-077 Barreiro, Portugal; 2 Faculty of Veterinary Medicine, University of Lisbon (FMV/ULisboa), Av. da Universidade Técnica, 1300-477 Lisbon, Portugal; 3 Interdisciplinary Centre for Research in Animal Health (CIISA), University of Lisbon (FMV/ULisboa), Av. da Universidade Técnica, 1300-477 Lisbon, Portugal; 4 Associate Laboratory for Animal and Veterinary Sciences (AL4AnimalS), 1300-477 Lisbon, Portugal; 5 Faculty of American Laser Study Club—ALSC, Altamonte Springs, FL 32714, USA; 6 Divisão de Medicina Veterinária, Guarda Nacional Republicana (GNR), Rua Cruz de Santa Apolónia, n° 16, 1149-064 Lisbon, Portugal; 7 Faculty of Veterinary Medicine, Lusófona University, 1749-024 Lisbon, Portugal; 8 Centro de Ciência Animal e Veterinária, Lusófona University, 1749-024 Lisbon, Portugal; 9 MED—Mediterranean Institute for Agriculture, Environment and Development, Instituto de Investigação e Formação Avançada, Universidade de Évora, Pólo da Mitra, Ap. 94, 7006-554 Évora, Portugal

The fields of small animal orthopedic surgery, physical therapy, and rehabilitation have undergone remarkable advancements, transforming the management of musculoskeletal conditions in companion animals [1,2]. This Special Issue is dedicated to exploring these innovations, highlighting collaborative research and practical applications that aim to enhance the quality of life for small animal patients.

Orthopedic conditions such as cranial cruciate ligament ruptures, patellar luxation, and degenerative joint diseases are common challenges in veterinary practice. Addressing these issues demands a multidisciplinary approach that combines surgical precision, therapeutic expertise, and ongoing care [3]. Innovations in minimally invasive techniques, such as arthroscopy, and the development of patient-specific implants through 3D printing have revolutionized surgical outcomes. Moreover, regenerative therapies, including platelet-rich plasma and stem cell treatments, offer promising avenues for restoring joint health and addressing chronic conditions such as osteoarthritis.

Rehabilitation has also become a cornerstone of comprehensive orthopedic care, shifting from a supplementary role to an essential part of treatment plans [4]. Evidence-based protocols incorporating hydrotherapy, neuromuscular electrical stimulation, and tailored therapeutic exercises are now integral to post-surgical recovery and the management of chronic conditions. These therapies not only accelerate recovery but also enhance mobility, particularly in aging animals or those with long-standing musculoskeletal challenges [5].

The articles featured in this Special Issue reflect the depth and breadth of progress in small animal orthopedic care. Studies include innovative approaches to planning patellar luxation surgeries, advancements in tibial plateau-leveling osteotomies, and biomechanical evaluations of implant techniques for complex cases [6,7,8,9,10]. Additionally, novel surgical interventions, such as the reconstruction of the quadriceps extensor mechanism and triceps brachii tendon [11,12], underscore the ingenuity required to address unique clinical presentations. Research on the effects of photobiomodulation and platelet-rich plasma on osteoarthritis and the impact of obesity on joint health further emphasizes the importance of preventive and therapeutic strategies in improving outcomes [13,14].

Beyond clinical techniques, this Special Issue highlights critical considerations in the broader care environment. For example, research on bacterial contamination in rehabilitation clinics underscores the importance of maintaining hygienic practices in these specialized facilities to safeguard patient health [15]. Together, these studies represent a cohesive effort to address the multifaceted challenges of small animal orthopedic and rehabilitation care.

The future of this field is promising, with continued advancements in technology, regenerative medicine, and precision surgery poised to redefine standards of care. Multidisciplinary collaboration will remain essential in integrating these innovations into practice, ensuring a holistic approach to diagnosis, treatment, and rehabilitation. By fostering ongoing dialogue and research, the veterinary community can continue to push the boundaries of what is possible, ultimately improving the lives of companion animals worldwide.

We extend our gratitude to the authors who contributed their expertise, the reviewers who ensured the rigor of the studies, and the editorial and MDPI management team for their dedication to presenting this collection. This Special Issue serves as both a reflection of progress and a call to action for further exploration and innovation in small animal orthopedics, physical therapy, and rehabilitation. Together, we can ensure that our patients lead healthier, more active, and pain-free lives.
